# Repeatability of Corneal Astigmatism and Equivalent Power with the MS-39 Tomographer Derived from Model Surface Fitting in a Cataractous Population

**DOI:** 10.3390/s25196171

**Published:** 2025-10-05

**Authors:** Achim Langenbucher, Nóra Szentmáry, Alan Cayless, Muntadher Al Karam, Peter Hoffmann, Theo G. Seiler, Jascha Wendelstein

**Affiliations:** 1 Department of Experimental Ophthalmology, Saarland University, 66424 Homburg, Germany; nszentmary@gmail.com (N.S.); wendelsteinjascha@gmail.com (J.W.); 2 Department of Ophthalmology, Semmelweis-University, Mária u. 39, 1085 Budapest, Hungary; 3 School of Physical Sciences, The Open University, Milton Keynes MK7 6AA, UK; alan.cayless@gmail.com; 4 Augenklinik am Universitätsspital Zürich, 8091 Zurich, Switzerland; muntadher.alkaram@usz.ch; 5 Augen- und Laserklinik Castrop-Rauxel, 44575 Castrop-Rauxel, Germany; pupillenpeter@gmx.de; 6 Institute for Refractive- and Ophthalmic Surgery (IROC), 8002 Zurich, Switzerland; theo@seiler.tv; 7 Department of Ophthalmology, Ludwig-Maximilians-University, 80336 Munich, Germany

**Keywords:** repeatability of astigmatism, repeatability equivalent power, astigmatic surface model fit, three-surface cornea model, epithelial mapping, corneal power vectors

## Abstract

**Highlights:**

**What are the main findings?**

- Modern high-resolution anterior segment tomographers are capable of extracting surface height data from the corneal front and back surfaces and from the epithelium–stroma interface.
- The higher refractive index of the corneal epithelium suggests that the cornea should be considered as a dual-layer structure to account for potential inhomogeneity in the epithelial thickness.

**What is the implication of the main finding?**

- Model surfaces, such as floating best-fit spheres or conoids, could be fitted to the height map data within a specific region of interest to determine relevant surface characteristics such as curvatures, asphericities, and apex positions.
- Based on a dataset with bilateral repeat measurements in a cataractous population, we were able to confirm that the extracted surface characteristics seem to be very robust. However, surface asphericity should be extracted from a larger region of interest to ensure more robust data.

**Abstract:**

We investigated the repeatability of the MS-39 in determining power vector components—the spherical equivalent (SEQ) and astigmatic powers (C0 and C45) and asphericity (Q)—of corneal epithelium, stroma, and endothelium in a large patient cohort. In this retrospective cross-sectional single-centre study, we evaluated a dataset containing 600 MS-39 anterior segment tomography measurements from 200 eyes (three repeat measurements each) taken prior to cataract surgery. The exported measurements included height map data for the epithelium, stroma, and endothelium surface. Model surfaces (spherocylinder (SphCyl), cylindrical conoid (CylConoid), and biconic (Biconic), all in the 3/6 mm zone) were fitted using nonlinear iterative optimisation, minimising the height difference between the measurement and model. The mean (MEAN) and standard deviation (SD) for each sequence of measurements were derived and analysed. In the 3 mm and 6 mm zone, the MEAN SEQ was 53.47/53.56/53.57 and 53.21/53.54/53.54 D for SphCyl/CylConoid/Biconic for the epithelium, −4.47/−4.51/−4.51 and −4.45/−4.50/−4.50 D for the stroma, and −6.23/−6.26/−6.26 and −6.18/−6.29/−6.30 D for the endothelium. With the three surface models and the 3/6 mm zone, the SD for SEQ/C0/C45 was in the range of 0.04 to 0.11/0.05 to 0.13/0.04 to 0.11 D for epithelium; 0.01 to 0.02/0.01 to 0.05/0.01 to 0.06 D for stroma; and 0.01 to 0.02/0.02 to 0.07/0.03 to 0.07 D for endothelium. Fitting floating model surfaces with astigmatism to map data of the corneal epithelium, stroma, and endothelium seems to be a robust and reliable method for extracting equivalent power and astigmatism using all the datapoints within a region of interest.

## 1. Introduction

The methods for extracting corneal astigmatism for use in the calculation of toric intraocular lenses include classical keratometry, corneal topography, and tomography [1,2,3,4]. Keratometry is based on a focal measurement of the corneal front surface curvature at two or more locations on two or more meridians in the midperiphery. Corneal (Placido disc-based) topography evaluates the corneal front surface curvature at thousands of measurement points, but is restricted to the evaluation of the midperipheral zone if simulated keratometry readings are requested. Finally, corneal tomography analyses the front and back surface curvature of the entire cornea but is again mostly restricted to an evaluation of the midperipheral zone of both corneal surfaces, similar to simulated keratometry, when corneal front and back surface readings are requested [2].

Modern corneal topographers and tomographers currently in use have excellent repeatability [1,2,3,5,6,7,8,9,10,11,12,13,14,15]. However, in situations with local irregularities, the curvature data derived from specific locations of the cornea might not fully represent the refractive properties of the cornea, with the consequence that lens power calculations might result in some prediction errors for the postoperative outcome [16]. The fitting of appropriate model surfaces to the tomographic data within a specific region of interest (ROI) might be a more robust alternative to extracting the curvature at specific corneal locations [16]. Where only corneal power values are required, simple model surfaces such as best-fit spheres or conoids would be sufficient. To allow for some potential decentration of the model surface with respect to the instrument axis, these surfaces require three additional degrees of freedom, namely axial shift (Z0) and lateral displacement in the horizontal (X0) and vertical (Y0) directions [16]. However, in order to take account of corneal astigmatism (for instance, in order to calculate toric implants), the surface models must be generalised to spherocylinders, cylindrical conoids, or biconic surfaces. The spherocylindrical model is based on a cornea with two (different) radii of curvature (R1 as the flat meridian located at axis A1 and R2 as the steep meridian orthogonally) without asphericity. The cylindrical conoid is based on a cornea with apical radii R1 at A1 and R2 orthogonally, together with a common asphericity (Q) in all corneal meridians. Finally, the biconic surface is based on a cornea with apical radii R1 with asphericity Q1 at meridian A1 and R2 with asphericity Q2 in the perpendicular meridian. Again, all of these model surfaces may be subject to some axial position Z0 and lateral displacement X0 and Y0 of the apex. R1 and R2 together with A1 could be used to determine the power of the corneal surface, and the asphericities could assist in the selection of the appropriate lens shape in terms of either a spherical design, or an aberration-neutral or aberration-correcting aspherical design.

Some modern tomographers based on high-resolution optical coherence tomography offer the option of measuring the interface between epithelium and stroma (stroma) in addition to the corneal front surface (epithelium) and back surface (endothelium) [17]. Since we know that the refractive index of corneal epithelium exceeds that of the stroma, we could consider the cornea as a three-surface model (duolayer) with three refractive surfaces, instead of a two-surface model (monolayer). This could be of significant interest, especially in situations where we might anticipate that the thickness of the epithelium may not be homogeneous. This could occur, for example, after laser vision correction for myopia or hyperopia. In such cases, restricting the analysis to a two-surface model could carry a significant risk of obtaining incorrect values for the corneal power.

The purpose of the present study was

- to extract height map data for the epithelium, stroma, and endothelium from a high-resolution anterior segment optical coherence tomographer,
- to develop a strategy for fitting floating spherocylinders, cylindrical conoids, and biconic surfaces to these height map data within a specific region of interest, and to extract the apical radii R1 and R2 together with the axis A1 and optionally the asphericity Q or Q1 and Q2,
- and using a large dataset of repeat measurements in a study population measured prior to cataract surgery to investigate the robustness of these parameters in order to quantify the repeatability of these characteristic metrics.

## 2. Materials and Methods

### 2.1. Dataset for Our Evaluation

The dataset considered in this study contained 3 repeat measurements for each of 206 eyes of 103 patients without a history of eye surgery (in total, N = 618 measurements). All measurements were taken prior to cataract surgery in patients scheduled for implantation of a non-toric intraocular lens. All measurements were performed at the IROC eye clinic (Zurich, Switzerland) with the MS-39 anterior segment optical coherence tomography device (CSO, Firence, Italy).

The measurement data were anonymised at source and exported as .CSV map files using the MS-39 software module for batch data export. For each measurement, a separate CSV file containing relevant patient data such as patient ID, the laterality (left or right eye), date of birth, sex (male or female), examination date and time, and map data with height data for the corneal epithelium, stroma, and endothelium, was generated. The map data were organised in cylindrical coordinates with 256 meridians in 31 concentric rings with a ring spacing of 0.2 mm (range from 0.0 to 6.0 mm). Invalid or unreliable data points within the map were indicated by a value of −1000.

Data tables were reduced to the relevant parameters required for our data analysis, consisting of the following measurements: patient ID and date of birth, exam date and time, the laterality (left or right eye), and height data for the corneal epithelium, stroma, and endothelium within the central 6 mm zone. The data were transferred to Matlab (Matlab, version 2024a, MathWorks, Natick, MA, USA) for further processing.

### 2.2. Data Pre-Processing in Matlab

Patient ages were calculated from the patient’s date of birth and the examination date. The following model surfaces were fitted to the map data of corneal epithelium, stroma and endothelium: (A) a floating spherocylinder (SphCyl) within a region of interest (ROI) of 3 mm (SphCyl3) and 6 mm in diameter (SphCyl6), both with 6 degrees of freedom: radius of curvature R1 in the flat meridian A1, radius of curvature R2 in the steep meridian, apex position in X (horizontally), Y (vertically) and Z (axially); (B) a floating cylindrical conoid (CylConoid) within a ROI of 3 mm (CylConoi3) and 6 mm in diameter (CylConoid6), both with 7 degrees of freedom: apical radius of curvature R1 in the flat meridian A1, apical radius of curvature R2 in the steep meridian, a common asphericity Q in all meridians, and apex position in X, Y and Z; and (C) a floating biconic surface (Biconic) within a region of interest of 3 mm (Biconic3) and 6 mm in diameter (Biconic6), both with 8 degrees of freedom: apical radius of curvature R1 in the flat meridian A1 with asphericity Q1, apical radius of curvature R2 in the steep meridian with asphericity Q2, and apex position in X0, Y0 and Z0 [16]. The steep meridian was assumed to be orthogonal to the flat meridian in all surface models. A surface fit was performed using nonlinear iterative optimisation techniques (SQP algorithm) based on minimising the root-mean-squared fit error in terms of height difference between the measurement height data and the height data of the surface model. The resulting parameters were specified in terms of the respective models (either SphCyl3_(.), SphCyl6_(.), CylConoid3_(.), CylConoid6_(.), Biconic3_(.) and Biconic6_(.)), followed by the surface (endothelium (Epi), Stroma (Stroma) or endothelium (Endo)) and the indicator for the fit parameter (.)R1, (.)R2, (.)A1, (.)Q, (.)Q1, (.)Q2, (.)X, (.)Y, (.)Z, respectively. Corneal thickness was extracted from the differences in the Z positions of the model surface apices: epithelial thickness was derived from (.)StromaZ – (.)EpiZ, stroma thickness from (.)EndoZ – (.)StromaZ, and total corneal thickness from (.)EndoZ – (.)EpiZ.

In the next step, we converted the radii R1 and R2 for each fit model and surface-to-surface power using literature data for the refractive indices for air (n = 1.0), epithelium (n = 1.41), stroma (n = 1.376), and aqueous humour (1.336). These power data were then decomposed, together with the orientations of the flat meridians A1, into power vector components: spherical equivalent power (SEQ), astigmatism projected to the 0° and 90° meridian (C0), and astigmatism projected to the 45° and 135° meridians.

In the last step, we derived the mean values for the power vector components (.)SEQ, (.)C0, (.)C45 and the asphericities (.)Q, (.)Q1, (.)Q2 for the sequence of 3 repeat measurements (indicated by (.)m) and the deviation of all repeat measurements from the mean of the 3 repeat measurements (indicated by (.)d), respectively.

### 2.3. Data Processing in Matlab and Statistics

The explorative statistics for the (.)m (per eye) and (.)d values of (.)R, (.)Q, (.)X, (.)Y and (.)Z (per measurement) are summarised in tables in terms of arithmetic mean, standard deviation, median, and the lower and upper boundaries of the 95% confidence interval (2.5% and 97.5% quantiles). Boxplots or raincloud plots are used for visualisation of the distributions of (.)SEQm, (.)Qm, (.)Q1m, (.)Q2m (the boxes refer to the interquartile range, and the whiskers to the 95% confidence interval), and double-angle plots showing C0m in the horizontal and C45m on the vertical axis are used for visualisation of the distributions of the astigmatic power vector components (.)C0m, (.)C45m. The bivariate astigmatic power vectors were analysed for bivariate normality using the Henze-Zirkler test [18]. In cases of bivariate normality, we calculated the centroids and the parametric error ellipses to display the 95% confidence ellipse for the scatter, and in cases of non-normality, we calculated the nonparametric medoids [19,20,21] and implemented iterative convex hull stripping techniques [22] to display the 95% confidence regions [23]. Since we expected mirror symmetry with respect to the facial axis (vertical axis), the double-angle plots are expected to show some symmetry for left and right eyes with respect to the horizontal axis (non-mirrored in C0 and mirrored in C45).

## 3. Results

From the N = 618 examinations of the N = 206 eyes of N = 103 patients where MS-39 measurements were transferred to us, and after considering the selection criteria, a dataset with N = 600 measurements (N = 200 eyes of N = 100 patients) was selected for our analysis (100 right and 100 left eyes of 57 female and 43 male patients). Three patients with a history of LVC were omitted from the dataset. The mean age of the patients was 67.2 ± 9.7 years (median 68.3 years, 95% confidence interval from 52 to 84 years).

Table 1 shows the mean values of the three repeat measurements in terms of radius of curvature data in the flat meridian ((.)R1) and in the steep meridian ((.)R2) for the surface fit with the spherocylindrical model; radius of curvature data in the flat meridian ((.)R1) and in the steep meridian ((.)R2) together with the common asphericity Q for the fit with the cylindrical conoid model; and radius of curvature ((.)R1) and asphericity data ((.)Q1) in the flat meridian and in the steep meridian ((.)R2 and (.)Q2) for the fit with the biconic model. The upper part of the table displays the surface fit within a region of interest, ROI = 3 mm, and the lower part displays the surface fit within a region of interest, ROI = 6 mm.

**Figure 1:**
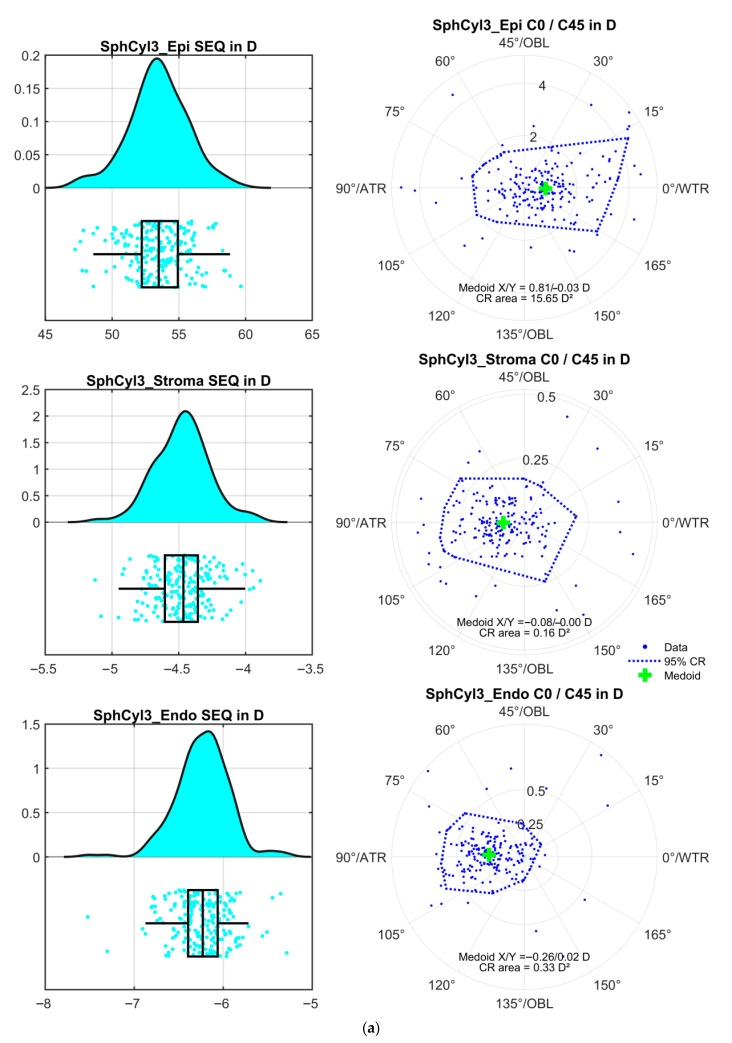

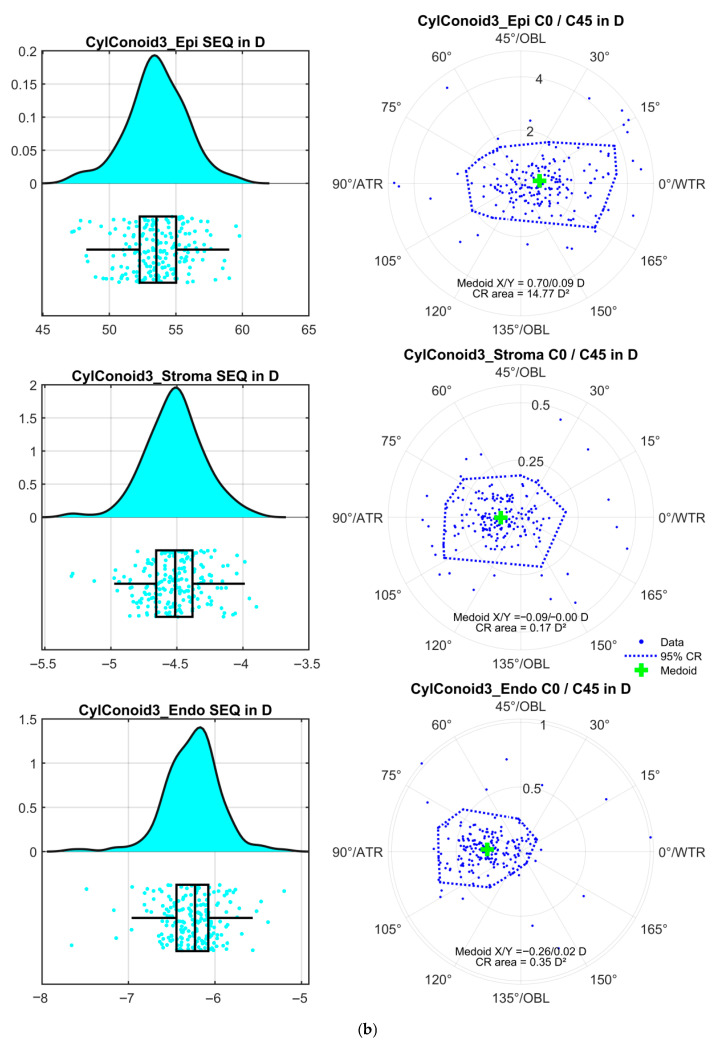

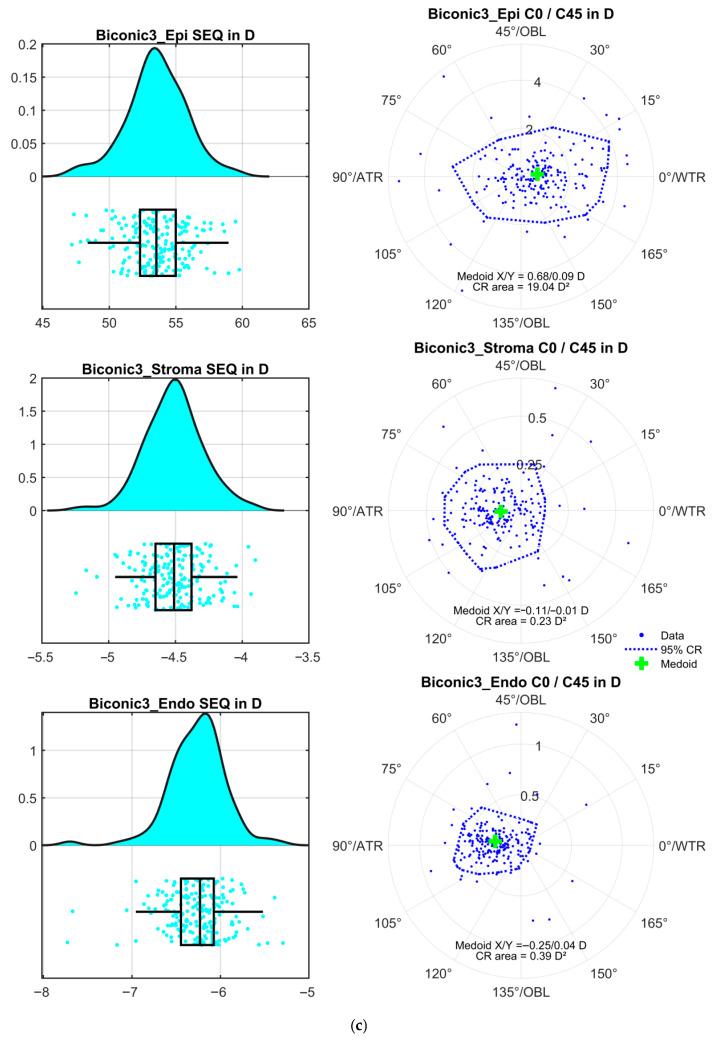
Distributions of the mean values of the power vector components as derived from the 3 repeat measurements for the corneal epithelium ((.)_Epi, upper graph), stroma ((.)_Stroma, middle graph), and endothelium ((.)_Endo, lower graph) within a region of interest of ROI = 3 mm. The graphs on the left display the probability density function PDF together with the boxplot and the data scatter (raincloud plot) for the spherical equivalent power SEQ. The double-angle plots on the right show the corresponding astigmatic power vector components (C0 in horizontal and C45 in vertical direction) together with the bivariate medoid and the 95% confidence region (CR) derived from iterative convex hull stripping. The coordinates of the medoid and the area of the CR are listed in the respective graphs. Subfigure (**a**) corresponds to the floating spherocylindrical surface model (SphCyl3_(.)), subfigure (**b**) to the floating cylindrical conoid surface (CylConoid3_(.)) and subfigure (**c**) to the biconic surface (Biconic3_(.)).

Figure 1 displays the distributions of the mean values of the power vector components derived from the three repeat measurements for the corneal epithelium (upper graph), stroma (middle graph), and endothelium (lower graph), for the surface fit within a region of interest of ROI = 3 mm. Figure 1a corresponds to the floating spherocylindrical surface model (SphCyl3_(.)), Figure 1b to the floating cylindrical conoid surface (CylConoid3_(.)) and Figure 1c to the biconic surface (Biconic3_(.)). The graphs on the left present raincloud plots for the spherical equivalent power SEQ, with the corresponding double-angle plots on the right. Since none of the bivariate distributions of the astigmatic power vector components exhibited normality, bivariate medoids and 95% confidence regions (CR) derived from iterative convex hull stripping are displayed in preference to centroids and 95% confidence ellipses. The coordinates of the medoids and the areas of the CRs are noted in each of the respective plots.

**Figure 2:**
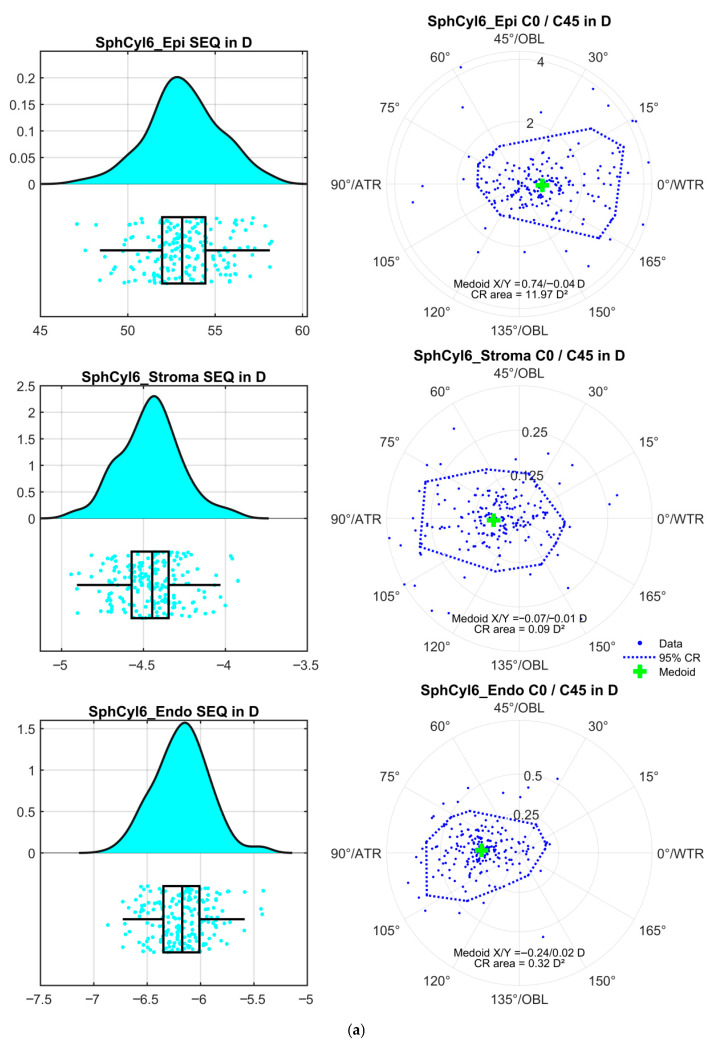

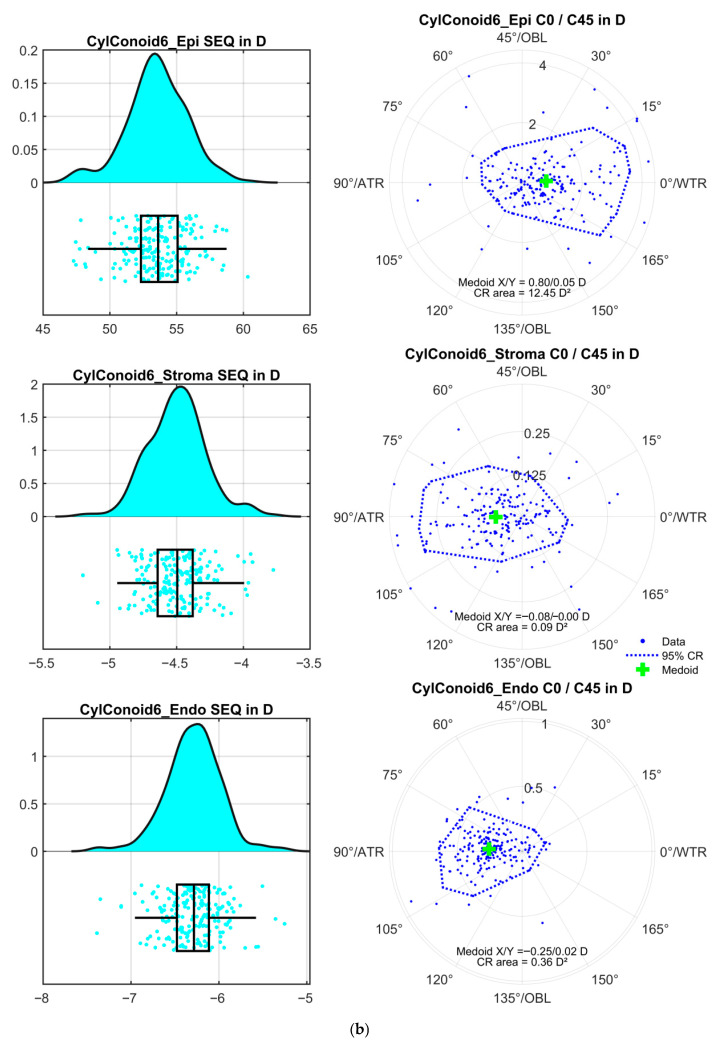

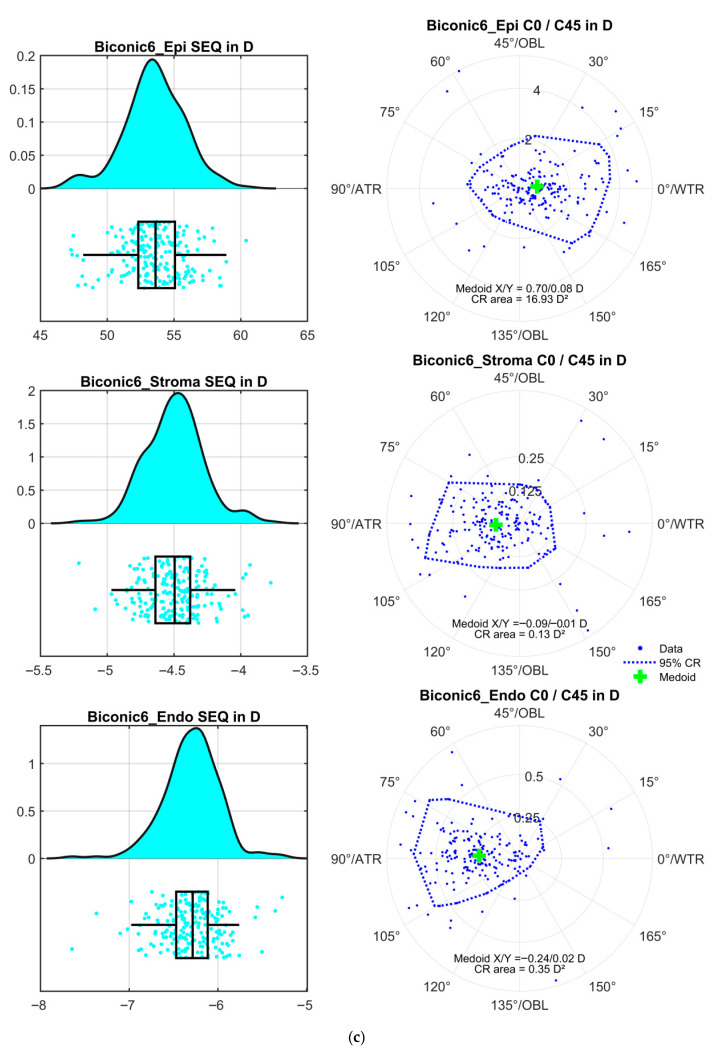
Distributions of the mean values of the power vector components as derived from the 3 repeat measurements for the corneal epithelium ((.)_Epi, upper graph), stroma ((.)_Stroma, middle graph), and endothelium ((.)_Endo, lower graph) within a region of interest of ROI = 6 mm. The graphs on the left display the probability density function PDF together with the boxplot and the data scatter (raincloud plot) for the spherical equivalent power SEQ. The double-angle plots on the right show the corresponding astigmatic power vector components (C0 in horizontal and C45 in vertical direction) together with the bivariate medoid and the 95% confidence region (CR) derived from iterative convex hull stripping. The coordinates of the medoid and the area of the CR are listed in the respective graphs. Subfigure (**a**) corresponds to the floating spherocylindrical surface model (SphCyl6_(.)), subfigure (**b**) to the floating cylindrical conoid surface (CylConoid6_(.)) and subfigure (**c**) to the biconic surface (Biconic6_(.)).

Figure 2 displays the corresponding distributions of the mean values of the power vector components derived from the 3 repeat measurements for the surface fit within a region of interest of ROI = 6 mm. Figure 2a corresponds to the floating spherocylindrical surface model (SphCyl6_(.)), Figure 2b to the floating cylindrical conoid surface (CylConoid6_(.)) and Figure 2c to the biconic surface (Biconic6_(.)). The graphs on the left present raincloud plots for the spherical equivalent power, SEQ, with the corresponding double angle plots on the right, together with the bivariate medoids and the 95% confidence regions.

Table 2 shows the deviations of surface model parameters from the mean values of the three repeat measurements in terms of radius of curvature data in the flat meridian ((.)R1) and in the steep meridian ((.)R2) for the surface fit with the spherocylindrical model; radius of curvature data in the flat meridian ((.)R1) and in the steep meridian ((.)R2) together with the common asphericity Q for the fit with the cylindrical conoid model; and radius of curvature ((.)R1) and asphericity data ((.)Q1) in the flat meridian and in the steep meridian ((.)R2 and (.)Q2) for the fit with the biconic model. The upper part of the table lists the values for the surface fit within a region of interest, ROI = 3 mm, and the lower part lists the values for the surface fit within a region of interest, ROI = 6 mm. The mean deviations all equal zero and are not listed in the table.

**Figure 3:**
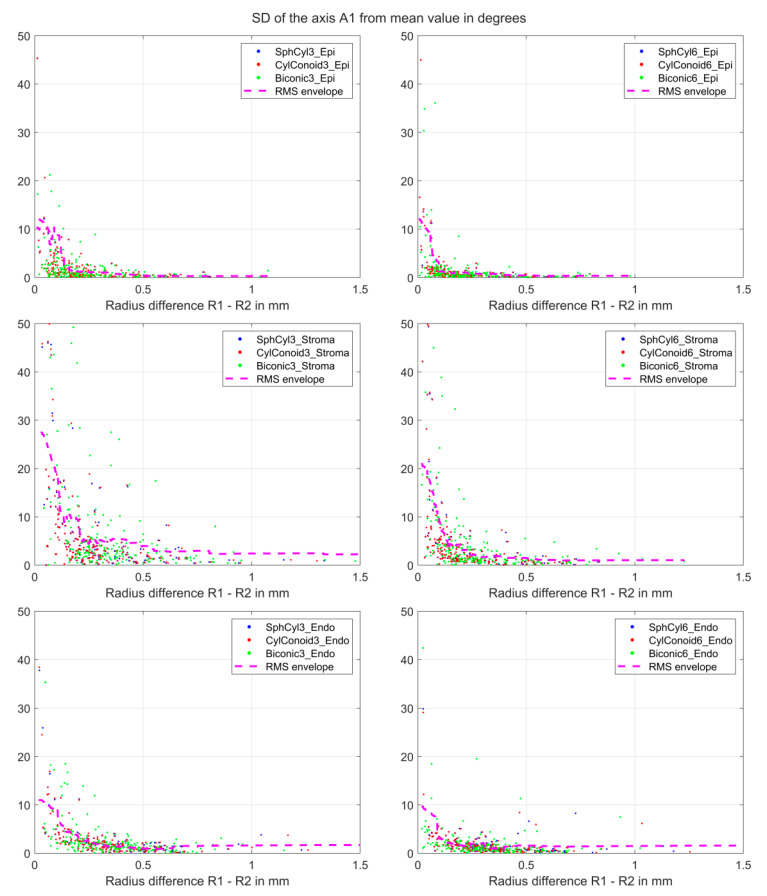
Standard deviation of the axis A1 (flat axis of the surface fit) for the 3 repeat measurements as a function of the radius difference in the flat and steep meridians for the corneal epithelium ((.)_Epi, upper graphs), stroma ((.)_Stroma, middle graphs), and endothelium ((.)_Endo, lower graphs). The left/right graphs display the situation with a surface fit within a region of interest ROI = 3 mm/6 mm. To show the general trend, we have added an envelope curve fitted to the root-mean-squared standard deviation values and merged the data of the 3 surface models together (magenta dashed line, N = 3·200 = 600 data points). It can be seen from this trend line that the uncertainty in A1 is systematically increased for low differences in the radii (corresponding to low astigmatism).

Figure 3 shows the standard deviation of the flat axis of the fitted surface model A1 for the three repeat measurements as a function of the radius difference in the flat and steep meridian for the corneal epithelium (upper graphs), stroma (middle graphs), and endothelium (lower graphs). The left/right graphs display the situation with a surface fit within a region of interest ROI = 3 mm/6 mm. The trend line is calculated as an envelope curve fitted to the root-mean-squared value of the standard deviations merged for all 3 surface models (magenta dashed line, N = 3·200 = 600 data points). It can be seen from this trend line that the uncertainty in A1 is systematically increased for low differences in the radii (corresponding to low astigmatism).

Table 3 lists the descriptive data for the thickness of the corneal epithelium (Epi), corneal stroma (Stroma), and total corneal thickness (Total, epithelium and stroma) derived from the surface fit with a floating spherocylinder (SphCyl), a cylindrical conoid (CylConoid) and a biconic surface (Biconic) within the 3 mm (SphCyl3, CylConoid3, Biconic3) or 6 mm region of interest (SphCyl6, CylConoid6, Biconic6). The upper part of the table shows the mean values of the three repeat measurements, and the lower part shows the deviations of the three repeat measurements from the respective mean value.

## 4. Discussion

In the last 3 decades, toric intraocular lenses have gained in popularity for the correction of corneal astigmatism. These are implemented either as classical toric lenses implanted in the capsular bag or as Add-On (piggy-back) lenses implanted in the sulcus ciliaris in front of a non-toric capsular bag lens. However, in either case, the calculation of toric lenses requires reliable data on corneal astigmatism. This could be derived from manual or automated keratometry, corneal topography, or tomography [23,24]. Some modern optical biometers already combine axial length measurement with topographic data (Placido topographer) or tomographic data (Scheimpflug or optical coherence tomography). A calculation strategy based only on corneal front surface data does not take into account the effect of the corneal back surface on the total corneal astigmatism, instead relying on statistical models or nomogram corrections, which may or may not represent the real corneal back surface astigmatism [2]. Corneal topographers are capable of providing measurement data of both corneal surfaces, enabling us to consider the cornea as a thick lens. However, the cornea is known to have a multilayer structure, with the most prominent layers being the epithelial and the stromal layers. Since the epithelium has a higher refractive index than the stroma, considering the cornea as a monolayer structure is always a simplification. This simplification to a monolayer might be sufficient for clinical applications such as lens power calculation if we could be confident that the epithelium was homogeneous in thickness, but in cases where the epithelium thickness profile is inhomogeneous (e.g., after laser vision correction), this simplification may be insufficient to represent the total corneal power or astigmatism [18].

The last decade has seen the introduction of new high-resolution anterior segment tomographers with highly relevant features such as epithelial mapping. Such tomographers have the potential to identify the interface between the corneal epithelium and stroma in addition to the corneal front and back surfaces. Measurement data from these instruments (e.g., for the surface height) can be exported directly as CSV maps organised in a Cartesian or cylindrical grid and postprocessed using custom software. However, there have not yet been any studies evaluating the reliability or repeatability of these map data in representing corneal power and astigmatism. For this purpose, we used a dataset derived from the modern high-resolution anterior segment tomographer MS-39 with three repeat measurements in both eyes of 100 patients to investigate this repeatability. Three different model surfaces were considered: a simple spherocylindrical surface restricted to the radii of curvature in both cardinal meridians, a cylindrical conoid which additionally provides a common asphericity value, and a biconic surface which includes separate asphericity values for both cardinal meridians [16]. These model surfaces were fitted to the height map data for the epithelium, stroma, and endothelium derived from the MS-39, both for a small region of interest (ROI = 3 mm) and for a larger region of interest (ROI = 6 mm). All model surfaces were considered as ‘floating surfaces’, meaning that the surface apex was not constrained to be located at the origin of the coordinate system [16]. Surface tilt (i.e., rotation with respect to the X and Y axes) was not considered in the current setup, but the model could be generalised to also include surface tilt (for the cylindrical conoid and biconic surface) [16]. However, in order not to overload this paper, we have not presented here the corresponding coordinates of the model surface apices. A surface fit was performed using a nonlinear iterative approximation strategy.

In the next step, we decomposed the apical radii of curvature together with the orientation of the flat axis into 3D power vector components, including the spherical equivalent power and the projection of astigmatism to the 0°/90° meridian and to the 45°/135° meridian [2,15]. The ‘mean surface model’ was derived from the three repeat measurements by averaging the corresponding power vector components and asphericities (this approach is preferable, since in this context, averaging the sphere, net astigmatism, and axis might be inappropriate). The deviations of the three repeat measurements from their mean values were then extracted as a measure of the variation or repeatability. Table 1 shows the mean values derived from the three repeat measurements for the model surface parameters, including radii of curvature and asphericity for the 3 model surfaces and the 3 surfaces of the cornea after fitting the surface to the map data within a 3 mm and a 6 mm ROI. Our results indicate that, on average, the radii of curvature are highest for the epithelium and lowest for the endothelium. As a result of the ‘normal’ negative asphericity of the cornea, the aspherical surface models (cylindrical conic and biconic surface) tend to yield slightly steeper apical radii as compared to the spherocylindrical model, and the surface fit within the ROI = 6 mm provides slightly flatter radii compared to the surface fit within the ROI = 3 mm for all surface models. The corresponding data for the deviations of the three repeat measurements from the mean values are listed in Table 2. We see that the repeatability of both the radii of curvature and the asphericity data is systematically higher for the epithelium compared to the stroma and endothelium for all surface models and both regions of interest. The within-subject standard deviation (standard deviation of the three repeat measurements, SD) is between 10 and 19 µm for the epithelium, 35 to 75 µm for the stroma, and 30 to 89 µm for the endothelium for the ROI = 3 mm fit zone, and between 6 and 22 µm for the epithelium, 20 to 35 µm for the stroma, and 14 to 26 µm for the endothelium for the ROI = 6 mm fit zone. This means that the surface fit in the larger ROI might be slightly more robust than that based on the smaller ROI. This superiority of the larger fit zone is even more pronounced for the asphericity values (Q, Q1, Q2), where the SD varies between 0.050 and 0.065 for ROI = 3 mm and between 0.023 and 0.051 for ROI = 6 mm. The thickness of the epithelium and stroma layer and the total corneal thickness extracted from the apices of the surface models are listed in Table 3. In accordance with literature data [6,8,12], the epithelium/stroma/total cornea shows an average thickness of 54 to 55 µm/486 µm/540 to 541 µm for all surface models and both ROIs. The within-subject standard deviation shows an excellent repeatability for the epithelium (6 to 7 µm), stroma (8 to 9 µm), and the total cornea (9 to 12 µm) for all surface models and both ROIs.

The mean power vector components as derived from the three repeat measurements of the apical radii of curvature and the orientation of the flat axis of the surface models are shown in Figure 2 for the 3 mm ROI and in Figure 3 for the 6 mm ROI. Our results indicate that the front surface shows a systematically higher power with the three-surface cornea model as compared to the two-surface cornea model. This is the result of the higher refractive index of the epithelium compared to the stroma. This leads to an epithelial surface power of 53 to 54 D compared to values known from the literature for the monolayer cornea, which generally range from 48 to 49 D. This high surface power is in part compensated by a negative surface power at the epithelium-stroma interface (−4 to −5 D) and by the corneal back surface power (−6 to −6.5 D). This means that with a three-surface model of the cornea, the front surface with the large step in the refractive index (from 1.0 to 1.41) is even more sensitive to any surface irregularity or asymmetry than expected from the two-surface model (1.0 to 1.376) or the 1 surface model (1.0 to 1.332 or 1.0 to 1.3375). The medoids stated in the double-angle plots on the right-hand graphs indicate that in our population, the epithelium shows a mean astigmatism with-the-rule of 0.68 to 0.81 D with all surface models and both ROIs, and that the epithelium-stroma interface shows a mean astigmatism of 0.07 to 0.11 D against-the-rule. The mean astigmatism of the corneal back surface (endothelium) matches quite well with the literature data, with a range of −0.24 to −0.26 D with all surface models and both ROIs, which could be subject to statistical or nomogram correction if only keratometric astigmatism is available. With the systematically larger step of the refractive index at the corneal front surface, it is obvious that the area of the confidence region (12 to 19 D^2^) is much larger compared to the area of the confidence region at the stroma (0.09 to 0.23 D^2^) or the endothelium (0.32 to 0.39 D^2^). This means that in a situation before cataract surgery (e.g., with implantation of a toric lens) if reliable data for the corneal power (especially the corneal astigmatism) are required [21,23], at least the corneal front surface measurements should be repeated and the centroid or medoid (power vector components) of the repeat measurements should be used, e.g., for (toric) lens power calculation. In contrast, for the corneal back surface (2 or 3 surface cornea model) and the stromal surface (3 surface cornea model), a single measurement seems to be sufficient for lens power calculation because the variation in the repeat measurements appears only to have a minor impact on the total corneal power.

However, the present study has some limitations: (A) we used a dataset with repeated measurements from an MS-39 anterior segment tomographer taken at a single centre. The results from a multicentre study or made using different anterior segment tomographers might differ slightly. (B) All measurements considered in this study are from a population scheduled for cataract surgery. The corresponding data in a younger study population or in eyes with corneal pathologies (e.g., ectatic diseases) may differ. (C) This study was restricted to 3 different surface models (spherocylinder, cylindrical conoid, and biconic) and 2 fit regions (ROI = 3 mm and 6 mm). Results based on other surface models (e.g., fringe Zernike surfaces [16]) might differ to some extent. (D) We used an iterative nonlinear strategy based on minimising the root-mean-squared height differences between the map data and the model surface height for fitting the model surface to the map data for the epithelium, stroma, and endothelium. Other fitting strategies may provide slightly different results.

## 5. Conclusions

The present study involved an investigation of the repeatability of the MS-39 tomographer. This is an example of a modern anterior segment tomographer as used for extracting the curvature, asphericity, and power vector components of the corneal epithelium, stroma, and endothelium surface. Height map data were exported with the standard software of the MS-39 and used to fit floating spherocylinder, cylindrical conoid, and biconic surfaces within two central regions of interest of diameters 3 and 6 mm. The mean values of three repeat measurements and the deviations of the repeat measurements from the mean values were assessed. The variation in the repeat measurements is systematically larger for the stroma and endothelium as compared to the epithelium, but the systematically larger step in the refractive index at the epithelium means that the variation in power vector components (spherical equivalent power and astigmatism projected to the 0°/90° and 45°/135° meridian) is systematically larger for the epithelium than for the endothelium and stroma. Where high reliability of spherical equivalent and astigmatic power is required (e.g., for (toric) lens power calculation), our recommendation is that repeat measurements should be made to ensure robust metrics, at least for the corneal front surface measurement, whereas for the corneal back surface and the epithelium-stroma interface, a single measurement with a high-resolution tomographer might be sufficient.

## Figures and Tables

**Table 1:** Mean values of the 3 repeat measurements with radius of curvature in the flat meridian (R1) and in the steep meridian (R2) together with the common asphericity (Q) and the asphericity in the flat (Q1) and the steep (Q2) meridians derived from a surface fit in the 3 mm zone (upper part) and in the 6 mm zone (lower part). Three different surface models were implemented: a floating spherocylinder (SphCyl, with parameters R1 and R2), a floating cylindrical conoid (CylConoid, with parameters R1, R2, and Q), and a floating biconic surface (Biconic, with parameters R1, R2, Q1, and Q2). The orientation of the flat axis is not shown, as the statistics of periodic axis values are not meaningful. All models were fitted to the epithelium, the stroma (interface between epithelium and stroma), and the endothelium as measured by the MS-39 anterior segment tomographer. SD refers to the standard deviation, and 2.5% quantile/97.5% quantile to the lower and upper boundaries of the 95% confidence interval.

Radii(R1, R2) [mm], Asphericity (Q, Q1, Q2) [1]		R1	R2	R1	R2	Q	R1	R2	Q1	Q2
**ROI 3 mm**		SphCyl3		CylConoid3			Biconic3			
Epithelium	Mean	7.8013	7.5671	7.7882	7.5538	−0.1685	7.7950	7.5493	−0.1526	−0.2290
	SD	0.3404	0.3286	0.3466	0.3358	0.2623	0.3475	0.3358	0.2759	0.3110
	Median	7.7778	7.5754	7.7581	7.5529	−0.1896	7.7490	7.5507	−0.1772	−0.2320
	2.5% quantile	7.1810	6.9388	7.1815	6.9222	−0.7001	7.1832	6.9144	−0.7342	−0.8694
	97.5% quantile	8.5964	8.3171	8.6126	8.3466	0.4119	8.6426	8.3370	0.4444	0.4510
Stroma	Mean	7.7704	7.4730	7.7056	7.4057	−0.3837	7.7463	7.3846	−0.3335	−0.4222
	SD	0.3845	0.3658	0.4002	0.3915	0.3210	0.3932	0.3891	0.3104	0.3066
	Median	7.7280	7.4601	7.6685	7.3894	−0.3640	7.7081	7.3675	−0.2926	−0.3674
	2.5% quantile	7.1605	6.7021	6.9875	6.5276	−1.1442	7.0316	6.5862	−1.0877	−1.0880
	97.5% quantile	8.7329	8.2779	8.6579	0.2534	0.3771	8.7334	8.2202	0.3268	0.3751
Endothelium	Mean	6.6194	6.2590	6.5972	6.2357	−0.2886	6.6008	6.2269	−0.2141	−0.2904
	SD	0.3367	0.3352	0.3515	0.3534	0.4297	0.3570	0.3500	0.4144	0.3612
	Median	6.5954	0.2519	6.5739	6.2389	−0.2414	6.5912	6.2347	−0.2448	−0.2971
	2.5% quantile	6.0315	5.6346	5.9935	5.4012	−1.1163	5.9880	5.3974	−1.0655	−1.0519
	97.5% quantile	7.4164	6.9361	7.4166	6.9009	0.5272	7.4329	6.8924	0.5338	0.3849
**ROI 6 mm**		SphCyl6		CylConoid6			Biconic6			
Epithelium	Mean	7.8261	7.6124	7.7809	7.5664	−0.1866	7.7895	7.5586	−0.1614	−0.2137
	SD	0.3232	0.3109	0.3441	0.3364	0.1854	0.3500	0.3354	0.2025	0.2069
	Median	7.8193	7.6253	7.7593	7.5606	−0.1948	7.7720	7.5597	−0.1796	−0.2043
	2.5% quantile	7.2420	7.0143	7.1393	6.9470	−0.5222	7.1448	6.9246	−0.4661	−0.7409
	97.5% quantile	8.4622	0.2677	8.6511	8.4193	0.2153	8.6390	8.3981	0.3803	0.1831
Stroma	Mean	7.7598	7.5330	7.6969	7.4689	−0.2466	7.7217	7.4455	−0.1568	−0.3331
	SD	0.3512	0.3219	0.3984	0.3743	0.2528	0.3972	0.3813	0.2378	0.2563
	Median	7.7473	7.5436	7.6746	7.4416	−0.2312	7.7019	7.4184	−0.1440	−0.3181
	2.5% quantile	7.1196	6.8844	7.0177	6.8457	−0.8834	7.0455	6.7393	−0.6605	−0.8961
	97.5% quantile	8.5927	8.2746	8.6746	8.4288	0.3078	8.7061	8.4090	0.3573	0.1217
Endothelium	Mean	6.6557	6.3320	6.5426	6.2133	−0.3187	6.5416	6.2100	−0.2962	−0.3433
	SD	0.2889	0.2800	0.3226	0.3374	0.2174	0.3258	0.3426	0.2603	0.2353
	Median	6.6369	6.3357	6.5227	6.1929	−0.3281	6.5238	6.1929	−0.3005	−0.3335
	2.5% quantile	6.1629	5.8419	6.9037	5.5799	−0.7444	5.9392	5.5858	−0.9739	−0.8602
	97.5% quantile	7.3289	6.9063	7.2769	6.8808	0.1680	7.2761	6.9015	0.2548	0.1078

**Table 2:** Deviations of the 3 repeat measurements from their mean value with radius of curvature in the flat meridian (R1) and in the steep meridian (R2) together with the common asphericity (Q) and the asphericity in the flat (Q1) and the steep (Q2) meridians derived from a surface fit in the 3 mm zone (upper part) and in the 6 mm zone (lower part). Three different surface models were implemented: a floating spherocylinder (SphCyl, with parameters R1 and R2), a floating cylindrical conoid (CylConoid, with parameters R1, R2, and Q), and a floating biconic surface (Biconic, with parameters R1, R2, Q1, and Q2). The mean deviations all equal zero and are not listed, and the orientation of the flat axis is not shown as it is not meaningful. All models were fitted to the epithelium, the stroma (interface between epithelium and stroma), and the endothelium as measured by the MS-39 anterior segment tomographer. SD refers to the standard deviation, and 2.5% quantile/97.5% quantile to the lower and upper boundaries of the 95% confidence interval.

Radii(R1, R2) [mm], Asphericity (Q, Q1, Q2) [1]		R1	R2	R1	R2	Q	R1	Q1	R2	Q2
**ROI 3 mm**		SphCyl3		CylConoid3			Biconic3			
Epithelium	SD	0.0112	0.0099	0.0167	0.0164	0.0555	0.0187	0.0174	0.0586	0.0637
	Median	0.0000	0.0002	0.0001	0.0000	0.0010	−0.0001	−0.0001	0.0000	0.0000
	2.5% quantile	−0.0219	−0.0215	−0.0347	−0.0360	−0.1131	−0.0412	−0.0377	−0.1123	−0.1139
	97.5% quantile	0.0216	0.0215	0.0320	0.0342	0.1117	0.0379	0.0337	0.1119	0.1135
Stroma	SD	0.0726	0.0348	0.0714	0.0467	0.0508	0.0754	0.0522	0.0544	0.0504
	Median	−0.0003	0.0001	0.0000	0.0010	0.0000	0.0003	0.0000	0.0000	0.0000
	2.5% quantile	−0.0396	−0.0429	−0.0512	−0.0732	−0.1117	−0.0786	−0.0773	−0.1132	−0.1108
	97.5% quantile	0.0444	0.0371	0.0560	0.0604	0.1124	0.0749	0.0784	0.1139	0.1102
Endothelium	SD	0.0875	0.0304	0.0892	0.0336	0.0652	0.0890	0.0377	0.0659	0.0629
	Median	−0.0007	0.0005	−0.0005	0.0008	0.0000	−0.0008	0.0000	0.0000	0.0017
	2.5% quantile	−0.0295	−0.231	−0.0356	−0.0359	−0.1135	−0.0510	−0.0506	−0.1139	−0.1142
	97.5% quantile	0.0230	0.0248	0.0335	0.0393	0.1145	0.0568	0.0556	0.1149	0.1141
**ROI 6 mm**		SphCyl6		CylConoid6			Biconic6			
Epithelium	SD	0.0061	0.0067	0.0094	0.0093	0.0228	0.0108	0.0106	0.0281	0.0312
	Median	−0.0001	0.0000	0.0000	0.0001	−0.0002	−0.0001	−0.0001	0.0001	−0.0005
	2.5% quantile	−0.0133	−0.0131	−0.0198	−0.0191	−0.0440	−0.0218	−0.0221	−0.0592	−0.0702
	97.5% quantile	0.0143	0.0134	0.0186	0.0214	0.0456	0.0215	0.0229	0.0589	0.0687
Stroma	SD	0.0204	0.0251	0.0261	0.0233	0.0350	0.0244	0.0250	0.0507	0.0493
	Median	−0.0002	0.0002	−0.0005	−0.0002	−0.0007	−0.0002	0.0002	−0.0001	0.0007
	2.5% quantile	−0.0202	−0.0162	−0.0281	−0.0347	−0.0753	−0.0426	−0.0397	−0.0961	−0.1054
	97.5% quantile	0.0204	0.0211	0.0329	0.0313	0.0742	0.0437	0.0372	0.1091	0.1021
Endothelium	SD	0.0139	0.0251	0.0257	0.0216	0.0238	0.0302	0.0226	0.0378	0.0348
	Median	0.0001	0.0002	−0.0004	−0.0002	0.0000	−0.0003	0.0000	−0.0001	−0.0003
	2.5% quantile	−0.0167	−0.0162	−0.0252	−0.0172	−0.0501	−0.0280	−0.0266	−0.0781	−0.0770
	97.5% quantile	0.0164	0.0211	0.0216	0.0208	0.0481	0.0264	0.0259	0.0827	0.0741

**Table 3:** Thickness of the corneal epithelium (Epi), corneal stroma (Stroma), and total corneal thickness (Total) derived from the surface fit with a floating spherocylinder (SphCyl), a cylindrical conoid (CylConoid) and a biconic surface (Biconic) within the 3 mm (SphCyl3, CylConoid3, Biconic3) or 6 mm region of interest (SphCyl6, CylConoid6, Biconic6). The upper part of the table lists the mean values of the 3 repeat measurements, and the lower part lists the deviations of the 3 repeat measurements from the corresponding mean value. SD refers to the standard deviation, and 2.5% quantile/97.5% quantile to the lower and upper boundaries of the 95% confidence interval.

Epithelial/Stromal/Total Corneal Thickness		Epi	Stroma	Total	Epi	Stroma	Total	Epi	Stroma	Total
Mean value of the 3 repeat measurements	ROI = 3 mm	SphCyl3			CylConoid3			Biconic3		
	Mean	0.0547	0.4856	0.5403	0.0546	0.4857	0.5402	0.0546	0.4856	0.5402
	SD	0.0044	0.0388	0.0389	0.0044	0.0388	0.0389	0.0044	0.0388	0.0389
	Median	0.0547	0.4834	0.5386	0.0546	0.4835	0.5384	0.0547	0.4835	0.5385
	2.5% quantile	0.0474	0.4083	0.4548	0.0474	0.4086	0.4551	0.0474	0.4086	0.4551
	97.5% quantile	0.0628	0.5538	0.6123	0.0627	0.5542	0.6125	0.0627	0.5542	0.6123
	ROI = 6 mm	SphCyl6			CylConoid6			Biconic6		
	Mean	0.0547	0.4864	0.5411	0.0546	0.4855	0.5401	0.0545	0.4855	0.5400
	SD	0.0043	0.0388	0.0390	0.0045	0.0388	0.0390	0.0045	0.0389	0.0390
	Median	0.0546	0.4854	0.5396	0.0545	0.4835	0.5384	0.0545	0.4835	0.5384
	2.5% quantile	0.0479	0.4076	0.4551	0.0469	0.4086	0.4547	0.0469	0.4086	0.4548
	97.5% quantile	0.0633	0.5578	0.6118	0.0626	0.5539	0.6123	0.0626	0.5555	0.6123
Deviation of the 3 measurements from the mean value	ROI = 3 mm	SphCyl3			CylConoid3			Biconic3		
	SD	0.0006	0.0008	0.0010	0.0006	0.0008	0.0009	0.0006	0.0008	0.0009
	Median	0.0000	0.0000	0.0000	0.0000	0.0000	0.0000	0.0000	0.0000	0.0000
	2.5% quantile	−0.0010	−0.0012	−0.0012	−0.0010	−0.0011	−0.0011	−0.0010	−0.0012	−0.0012
	97.5% quantile	0.0010	0.0014	0.0013	0.0010	0.0012	0.0013	0.0010	0.0013	0.0013
	ROI = 6 mm	SphCyl6			CylConoid6			Biconic6		
	SD	0.0007	0.0009	0.0012	0.0007	0.0008	0.0011	0.0007	0.0009	0.0012
	Median	0.0000	0.0000	0.0000	0.0000	0.0000	0.0000	0.0000	0.0000	0.0000
	2.5% quantile	−0.0009	−0.0014	−0.0012	−0.0010	−0.0014	−0.0012	−0.0010	−0.0015	−0.0013
	97.5% quantile	0.0010	0.0014	0.0012	0.0010	0.0014	0.0013	0.0010	0.0015	0.0013

## Data Availability

Data could be provided upon request to the authors.
